# Telmisartan Inhibits Cell Proliferation by Blocking Nuclear Translocation of ProHB-EGF C-Terminal Fragment in Colon Cancer Cells

**DOI:** 10.1371/journal.pone.0056770

**Published:** 2013-02-22

**Authors:** Keiji Ozeki, Satoshi Tanida, Chie Morimoto, Yoshimasa Inoue, Tsutomu Mizoshita, Hironobu Tsukamoto, Takaya Shimura, Hiromi Kataoka, Takeshi Kamiya, Eiji Nishiwaki, Hiroshi Ishiguro, Shigeki Higashiyama, Takashi Joh

**Affiliations:** 1 Department of Gastroenterology and Metabolism, Nagoya City University Graduate School of Medical Sciences, Nagoya, Aichi, Japan; 2 Department of Living Science Nutrition Course, Matsuyama Shinonome Junior College, Matsuyama, Ehime, Japan; 3 Carna Biosciences Incorporation, Kobe, Japan; 4 Department of Medical Technology School of Health Sciences, Gifu University of Medical Science, Seki, Gifu, Japan; 5 Department of Cell Growth and Tumor Regulation, Proteo-Medicine Research Center, Ehime University, Toon, Ehime, Japan; 6 Department of Biochemistry and Molecular Genetics, Ehime University Graduate School of Medicine, Ehime University, Toon, Ehime, Japan; Shanghai Jiao Tong University School of Medicine, China

## Abstract

**Background & Aims:**

Current treatment target toward advanced colorectal cancers is mainly focused on the epidermal growth factor receptor (EGFR) signaling, but its additive effects with chemotherapy are still limited. A disintegrin and metalloproteinase (ADAM) cleaves the proheparin-binding epidermal growth factor like growth factor (proHB-EGF). And soluble HB-EGF activates EGFR. In parallel, the carboxy-terminal fragment of proHB-EGF (HB-EGF-CTF) translocates into the inner nuclear membrane, and subsequently exerts on the regulation of cell proliferation by binding nuclear promyelocytic leukemia zinc finger (PLZF) protein, a transcriptional repressor, thereby causing its nuclear export. We hypothesized that the inhibition of HB-EGF-CTF nuclear translocation may be a new strategy in preventing cell proliferation.

**Methods:**

12*-O-*tetradecanoylphorbor-13-acetate (TPA) was treated to activate ADAM. Nine-thousand chemical compounds were screened for their efficacies in blocking the binding of HB-EGF-CTF to promyelocytic leukemia zinc finger (PLZF) with Alphascreen system. The obtained candidates were then used to block the binding of HB-EGF-CTF to PLZF in colon cancer cells, HT29 and HCT116. Cell proliferation was investigated with a growth curve assay. The intracellular localization, and association between HB-EGF-CTF and PLZF, was assessed with immunofluorescent staining, and immunoprecipitation and Western blotting, respectively. The effects of obtained candidates on EGFR phosphorylation and on nuclear translocation of HB-EGF-CTF and export of PLZF during the angiotensin II type1 receptor (AT1R) knockdown were also investigated.

**Results:**

Telmisartan and candesartan were found to be potential candidates. Telmisartan inhibited TPA-induced cell proliferation stronger than candesartan. Telmisartan, but not candesartan blocked the nuclear translocation of HB-EGF-CTF, and binding of HB-EGF-CTF to PLZF, during TPA stimulation. Both telmisartan and candesartan did not inhibit TPA-induced EGFR phosphorylation, and telmisartan, but not candesartan, inhibited TPA-induced nuclear translocation of HB-EGF-CTF after knockdown of AT1R.

**Conclusions:**

The inhibition of HB-EGF-CTF nuclear translocation with telmisartan may be a novel strategy in preventing cell proliferation.

## Introduction

Colorectal cancer is the leading cause of death from gastrointestinal malignancy [1]. Currently therapeutic treatments toward advanced colorectal cancers are mainly focused on the design of therapeutic agents that is targeted to the epidermal growth factor receptor (EGFR), the monoclonal blocking antibodies such as cetuximab and panitumumab [2], [3]. The additive effects of these therapeutic antibodies plus combination chemotherapy on advanced colorectal cancer are obtained, but limited because the KRAS mutation abolished these effects [3], [4].

N-terminal fragments of EGFR ligands including heparin-binding epidermal growth factor like growth factor (HB-EGF) which is considered to be involved in mainly cell proliferation in colon cancer cells bind to EGFR and induce its phosphorylation [5], [6], [7], [8], [9]. G-protein coupled receptor (GPCR) agonists (e.g. interleukin-8) and 12-*O*-tetradecanoylphorbol-13-acetate (TPA) induced EGFR transactivation through a disintegrin and metalloproteinase (ADAM) - cleaved proHB-EGF [10], [11]. The carboxy -terminal fragments of proHB-EGF (HB-EGF-CTF) produced by ectodomain shedding translocate into the inner nuclear membrane, subsequently exert on the regulation of cell proliferation by binding nuclear promyelocytic leukemia zinc finger (PLZF) protein, a transcriptional repressor, thereby causing its nuclear export [12], [13]. Therefore, inhibition of nuclear translocation signaling of HB-EGF-CTF is considered to be a new strategy against the development of colorectal cancer. However, there is no evidence of therapeutic agents that specifically block nuclear translocation of HB-EGF-CTF and binding of that to PLZF.

Thus, the aims of the present study were to: i) screen for potent chemical compounds that are capable of inhibiting the interactions between HB-EGF-CTF and PLZF via GST-pull down assay, surface plasmon resonance (SPR) spectroscopy and Alphascreen technology, ii) validate the inhibitory effects of these potent compounds on cell proliferation, and iii) certify the importance of inhibiting the nuclear translocation of HB-EGF-CTF in colon cancer cell proliferation.

## Materials and Methods

### Materials

TPA was purchased from Cell Signaling Technology (Berverly, MA, USA). Anti-EGFR polyclonal antibody, anti-phosphotyrosine monoclonal antibody (4G10) and normal IgG were purchased from Millipore (Billerica, MA, USA). The EGFR tyrosine kinase inhibitor AG1478 and anti-PLZF antibody were purchased from Calbiochem (LA Jolla, CA, USA). Candesartan was obtained from Takeda Pharmaceutical Company Limited (Osaka, Japan) and telmisartan was a kind gifted from Boehringer Ingelheim (Ingelheim, Germany). Olmesartan and losartan were purchased from Daiichi Sankyo Company limited (Tokyo, Japan) and Merck Sharp & Dohme (Whitehouse Station, NJ, USA), respectively. The anti-HB-EGF-CTF polyclonal antibody [12] and metalloproteinase inhibitor, KB-R7785 [14], were prepared. The monoclonal anti-FLAG M2 antibody and the peroxisome proliferator-activated receptor (PPAR) γ antagonist, GW9662 [15], [16], were purchased from Sigma-Aldrich (St. Louis, MO, USA).

### Plasmid Preparation

A plasmid encoding FLAG-tagged PLZF was generated by subcloning the FLAG sequence and human PLZF cDNA into pcDNA3.1 (Invitrogen, Carlsbad, CA, USA). Plasmids for the recombinant expression of FLAG-tagged PLZF derivatives, GST-fused EGFR ligands-CTF, and CFP (cyan fluorescein protein)-tagged PLZF were prepared [12]. Zinc fingers (ZnF) 5-8, and, EGFR-CTF were cloned into pGEX6P-1(GE Healthcare, Little Chalfont, U.K.).

### Cell Culture

Human colon cancer cell lines, HT29 and HCT116 (American Type Culture Collection, Rockville, MD, USA), were cultured with McCoy’s5A (Sigma), CaCo2 (American Type Culture Collection) were cultured with Dulbecco's modified Eagle's medium (DMEM) with 20% fetal bovine serum (FBS). And SW480 (American Type Culture Collection) was cultured DMEM with 10% FBS. HT1080 cells and primary human keratinocytes were cultured [12], [17].

### GST Pull-down Assay

GST and GST-fused proHB-EGF-CTF, GST- transforming growth factor-α (TGF-α)-CTF, GST- amphiregulin (AR)-CTF, and GST- epiregulin (EPR)-CTF were prepared [12]. After binding GST and GST-EGFR ligands-CTF to the glutathione Sepharose beads, lysates containing various FLAG-tagged PLZF derivatives were incubated with 20 µL of beads for 2 h at 4°C. After washing, bound proteins were analyzed with immunoblotting using an anti-FLAG antibody.

### Surface Plasmon Resonance (SPR) Spectroscopy

A BIA core S51 SPR system® (GE Health care) was used to qualitatively and quantitatively analyze the interactions between the four immobilized EGFR-ligand-CTFs (i.e. HB-EGF-CTF, TGF-α-CTF, AR-CTF, and EPR-CTF) and GST-Zn 5-8 of PLZF, which were measured in resonance units (RU). The recombinant biotin-EGFR- ligand-CTFs (Peptide Institute, Inc., Osaka, Japan) were individually immobilized (∼1000 RU) through amino group coupling on streptavidin (SA) certified chips. Various concentration of GST-Zn5-8 were added into running HEPES buffer (10 mM HEPES, pH 7.4, 150 mM NaCl, 0.005% Surfactant P20) at a flow rate of 30 µL/min for 2 min. The sensor chips were dissociated by a 30-sec injection of 10 mM Glycine-HCl, pH 1.5. The sensorgrams were analyzed with BIA evaluation Software (version 4.1; BIAcore). The equilibrium dissociation constants ('binding constant'; KD) of each reaction were calculated by dividing the dissociation rates ('off rate'; kd) by the association rates ('on rate'; ka) (i.e. Kd = kd/ka).

### Imaging of CFP Fusion Proteins and Quantitation of Cellular Fractions with Nuclear Localized CFP-PLZF

Transiently transfected HT1080 cells, and stable HT1080/HB-EGF, HT1080/TGF-α, HT1080/AR, and HT1080/EPR cells were cultured for 24 h and then cells were pretreated with 10 µM KB-R7785 in serum-free medium for 30 min. Cells were then incubated in serum-free medium with 100 nM TPA for 1 h. Subcellular localization of CFP fusion protein was examined under an epifluorescence microscope (Eclipse TE3000, Nikon, Tokyo, Japan) [12].

### Alphascreen Assays

An Alphascreen system® (PerkinElmer, Shelton, CT, USA) was used to screen a library of 9000 chemical compounds (Carna Bioscience Inc., Kobe, Japan) cyclopaedically for their efficacy in blocking the interaction between biotinylated-EGFR ligands-CTF and GST-Zn5-8 of PLZF. 5 µl of 1.25 µg/mL recombinant GST-ZnF5-8 was incubated with 2.5 µL of 1.25 µg/mL biotin-EGFR ligands-CTF for 30 min, and 2.5 µL of anti-GST antibody (1.8 µg/mL) was added for over 1 h. Then 5 µL streptavidin-coated donor and anti-GST IgG conjugated acceptor beads (1∶1 mixture) were incubated for 2 h in the dark. Alphascreen signals (counts per second) were analyzed with an EnVision microplate reader (Perkin-Elmer). Assays were conducted at 23°C in buffer containing 25 mM HEPES, 1 mM MgCl_2,_ 20 mM NaCl, pH 7.4, 0.1% BSA.

### Cell Proliferation Assay (CCK-8 Kit Assay)

A Cell Counting Kit8 (CCK-8) (Dojindo Laboratories, Kumamoto, Japan) was used for determining cell proliferation. Briefly, 2×10^5^ cells per well were cultured in 96-well culture plates with 10% FBS for 24 h. Then, FBS was replaced with serum free medium for 72 h with or without 30 µM of the 12 candidate compounds and telmisartan or candesartan (0.3, 3 and 30 µM), and 30 µM of GW9662 during stimulation with 100 nM of TPA. 10 µL of CCK-8 solution was then added to each well, and incubated at 37°C for an additional 2 h. Optical density (OD) was examined at a wave-length of 450 nm.

### Monitoring Single Cell Growths

Cells were seeded at a density of 5×10^2^ cells per 10 cm of a dish on day 0 in 10% FBS normal growth medium. After 24 h (on day1), fairly isolated and morphologically healthy cells were marked with phase-contrast microscopy. The medium was replaced with 5% FBS medium (control) with or without 100 nM of TPA, 10 µM of KB-R7785, 100 nM of AG1478, 30 µM of telmisartan and candesartan, and 50 ng/mL of recombinant HB-EGF (Sigma). The medium was exchanged every 48 h with fresh medium. Cell morphology and counts were scored every 24 h [18].

### Immunofluorescence Microscopy

Cells that formed a colony were switched to serum-free medium for 48 h and then incubated for 60 min in 5%FBS conditioned media with or without 10 µM of KB-R7785, 100 nM of AG1478, 50 ng/mL of recombinant HB-EGF, 30 µM of telmisartan, and 30 µM of candesartan. Subsequently, cells were treated with 100 nM of TPA for 90 min, and then fixed with ethanol and acetone. The subcellular localization of HB-EGF-CTF and PLZF was analyzed with immunofluorescence. Primary antibodies against HB-EGF-CTF or PLZF were used. The secondary antibodies were Alexa Fluor 594 anti-rabbit IgG or Alexa Fluor anti-mouse IgG (Invitrogen). Images were obtained on an Eclipse 80i fluorescence microscope (Nikon).

### Immunoprecipitation and Western Blotting

Cells in a subconfluent state were switched to serum-free medium for 48 h and treated with TPA at the indicated times. Telmisartan and candesartan were added to the cells for 60 min, prior to the stimuli. Cells were lysed with lysis buffer, and supernatants were collected and incubated with 1 µg of human anti-EGFR or anti-HB-EGF-CTF polyclonal antibodies for 2 h at 4°C with end-over-end rotation. Immunoprecipitation and Western blotting were performed [11]. The primary antibodies used were 4G10 or anti-PLZF, and anti-HB-EGF-CTF. The secondary antibodies used were anti-mouse IgG HRP-linked or anti-rabbit IgG HRP-linked antibodies (Cell Signaling Technology). The membranes were developed on an ECL Western blotting detection system (Amersham Biosciences, Buckinghamshire, England).

### Silencing the Angiotensin II type 1 Receptor (AT1R) and ADAM12

Cells were transfected with 30 µM of AT1R and scramble siRNAs (Santa Cruz, Delaware, CA, USA), and with 10 µM of ADAM12 and scramble siRNAs (Invitrogen) using lipofectamine reagent (Invitrogen). 72 h after cells were transfected, the expression of AT1R and ADAM12 (Santa Cruz Biotechnology) were analyzed by Western blotting.

### Statistics

Data are expressed as mean± SEM. The one-way ANOVA and Turkey-Kramer’s multiple comparison procedure test were used to determine statistically significant differences (P<0.05).

## Results

### Dual Signaling Pathways of EGFR Phosphorylation and HB-EGF C-terminal Fragment Nuclear Translocation during Cell Proliferation (Quoted from Ref No. [19] and Modified.)

**Figure 1:**
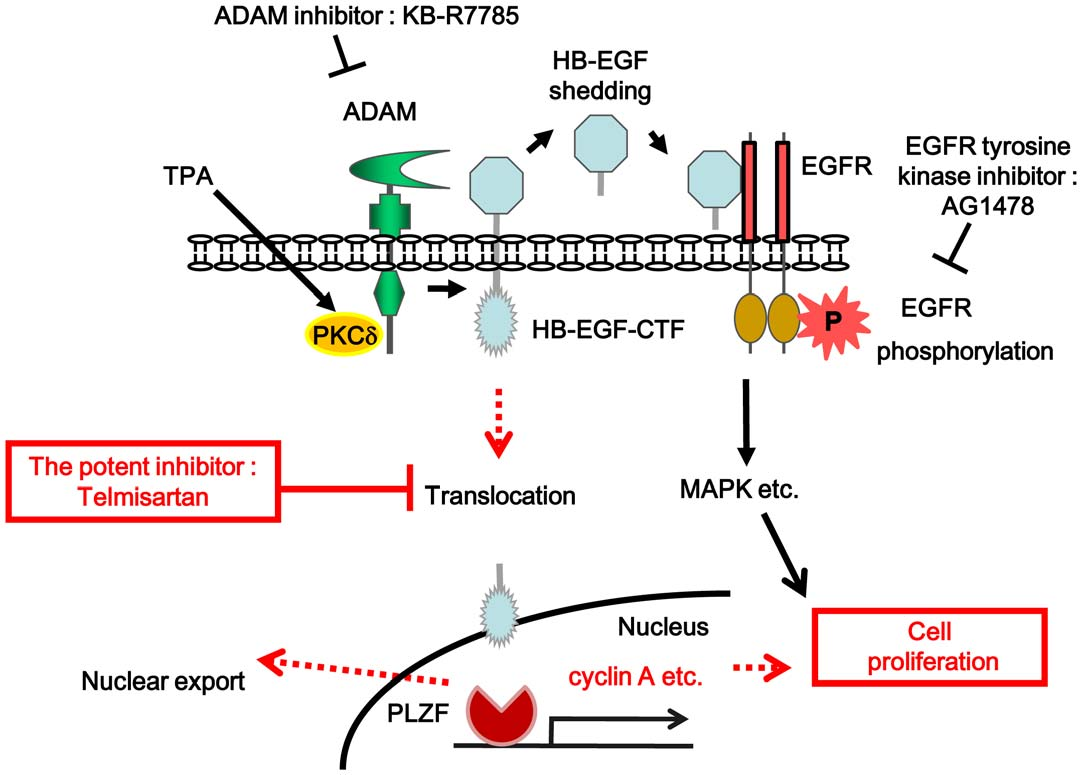
Dual signaling pathways of EGFR phosphorylation and HB-EGF C-terminal fragment nuclear translocation during cell proliferation. Quoted from [19] and modified. TPA induces an ADAM-mediated cleavage of proHB-EGF, and results in the ectodomain shedding of its N-terminal fragment and generation of an intracellular C-terminal fragment (CTF). The soluble HB-EGF binds to the EGFR and induces a rapid transient phosphorylation of EGFR. This phosphorylation results in the transcription of various genes. Meanwhile the HB-EGF-CTF is translocated into the nucleus, where it subsequently induces the nuclear export of PLZF. This results in the progression of cell cycle. The potent inhibitor blocks the nuclear translocation of HB-EGF-CTF. P indicates phosphorylation. Abbreviations: EGFR; epidermal growth factor receptor, TPA; 12-*O*-tetradecanoylphorbol-13-acetate, PKCδ; protein kinase Cδ, ADAM; a disintegrin and metalloproteinase, HB-EGF; heparin-binding EGF-like growth factor, CTF; C-terminal fragment, MAPK; mitogen-activated protein kinase, PLZF; promyelocytic leukemia zinc finger.

12-*O*-tetradecanoylphorbor-13-acetate (TPA) induced epidermal growth factor receptor (EGFR) transactivation through a disintegrin and metalloproteinase (ADAM) - cleaved proheparin-binding EGF-like growth factor (proHB-EGF) and regulates cell proliferation [10]. In parallel, the carboxy-terminal fragments (CTF) of proHB-EGF (HB-EGF-CTF) translocate into the inner nuclear membrane, subsequently exert on the regulation of cell proliferation by binding nuclear promyelocytic leukemia zinc finger (PLZF) protein, a transcriptional repressor, thereby causing its nuclear export (Fig. 1) [19].

We hypothesized that inhibition of nuclear translocation of HB-EGF-CTF might lead to a new strategy for prevention cell proliferation during colon cancer development.

However, there is currently no evidence of potent candidates that specifically block the nuclear translocation signaling of HB-EGF-CTF.

**Figure 2:**
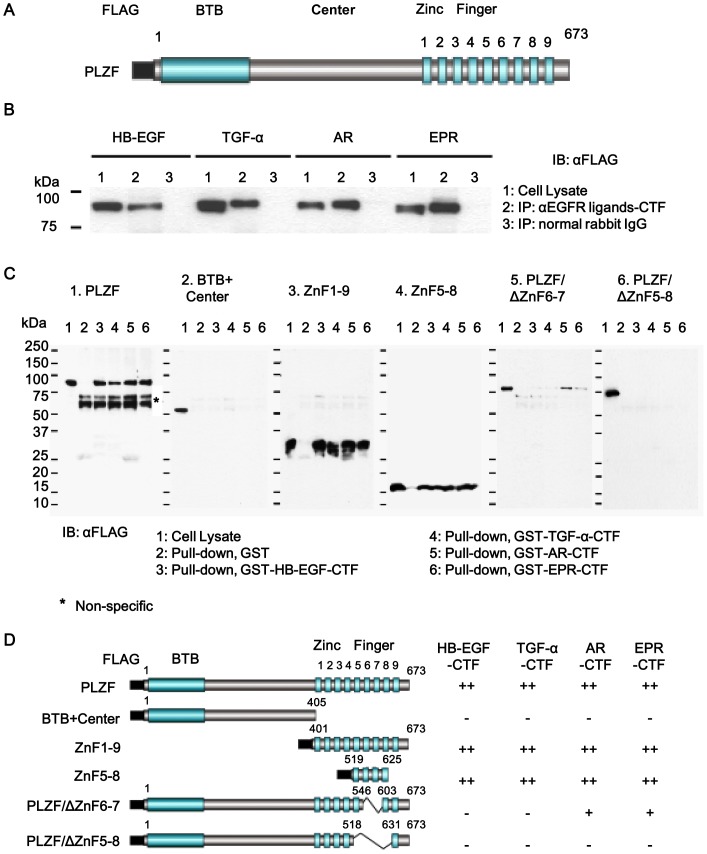
Binding of EGFR ligand-CTFs to ZnF5-8 region of PLZF. (A) Schema of FLAG-tagged full length PLZF consisting of FLAG, BTB, Center, and nine ZnFs. (B) HT1080 cells stably expressing pro-HB-EGF, pro-TGF-α, pro-AR and pro-EPR were transiently transfected with an expression vector encoding FLAG-tagged PLZF. PLZF protein expression of cell lysates (lane1), as well as, cells treated with 100 nM TPA for 1 h, and probed with anti-FLAG antibodies following immunoprecipitation with anti-EGFR ligands-CTF antibodies (lane2) and an anti-normal rabbit IgG (lane3). (C) GST pull-down assay. Cell lysates containing FLAG-tagged PLZF derivatives were incubated with GST (lane 2), GST-HB-EGF-CTF (lane 3), GST-TGF-α-CTF (lane4), GST-AR-CTF (lane 5), and GST-EPR-CTF (lane 6) beads for 2 h, and bound proteins were detected by immunoblotting with an anti-FLAG antibody. (D) Schema of FLAG-tagged PLZF derivatives. The binding properties of PLZF derivatives to GST-fused- HB-EGF-CTF, TGF-α-CTF, AR-CTF and EPR-CTF GST in a pull-down assay, as summarized in the right lanes of each structure. Binding properties are based on the estimation of band intensity with are relative to the control band and indicated by++(>50%),+(50–10%), and − (<10%).

**The CTF of EGFR ligands interacted with the specific part of PLZF (ZnF5-8)**We used the HT1080 cell lines because HT1080 cells express little endogenous HB-EGF precursors, and they are helpful to analyze the binding sites between HB-EGF-CTF and PLZF, and nuclear export of PLZF after overexpressing HB-EGF precursors and PLZF derivatives [12]. We first certified the interaction between the cytoplasmic domains of EGFR ligands and PLZF in HT1080 cells expressing the four proEGFR-ligands and PLZF before screening the potent candidates. Given that PLZF coimmunoprecipitates with an antibody against the CTF of proHB-EGF in HT1080 cells overexpressing proHB-EGF and FLAG-tagged PLZF [12], a coimmunoprecipitation of PLZF was performed with antibodies against the CTF of the four EGFR ligands (HB-EGF, TGF-α, AR, EPR), in HT1080 cells. The FLAG-tagged full length PLZF gene was transiently transfected into cells stably expressing proHB-EGF, proTGF-α, proAR or proEPR (Fig. 2A). PLZF was expressed in these cells (Fig. 2B, lane 1). After TPA stimulation for 1 h, FLAG-tagged PLZF protein which immunoprecipitated with each antibody was detected by immunoblotting with an anti-FLAG antibody (Fig.2B, lane 2).

Next, we determined the binding regions of PLZF to the cytoplasmic domains of pro-EGFR ligands in HT1080 in the GST pull-down assay. A GST pull-down assay showed the interaction between the EGFR ligand-CTFs and ZnF 5-8 of PLZF. Each recombinant GST-fused CTF (GST-CTF) pulled down FLAG-tagged PLZF equally in cell lysates prepared from HT1080 cells expressing FLAG-tagged PLZF (Fig. 2C, panel 1). We further investigated the binding regions of the CTFs in PLZF. Various FLAG-tagged PLZF derivatives (Fig. 2D) were prepared and incubated with recombinant GST-EGFR ligand-CTFs. The whole zinc finger region (ZnF1-9) and ZnF5-8 were pulled down equally by GST-TGF-α-CTF, GST-AR-CTF and GST-EPR-CTF as well as HB-EGF-CTF, but not GST alone (Fig. 2C, panels 3 and 4). None of the four GST-CTFs pulled down the deletion mutant lacking a zinc finger region (BTB+Center) (Fig. 2C, panel 2). We then tested whether the deletion mutants of ZnF6-7 (PLZF/ΔZnF6-7) and ZnF5-8 (PLZF/ΔZnF5-8) bind the GST-CTFs. The deletion of ZnF6-7 abrogated the binding of PLZF to GST-TGF-α-CTF and GST-HB-EGF-CTF, suggesting that TGF-α-CTF and HB-EGF-CTF interact with PLZF via ZnF6-7. In contrast, PLZF/ΔZF6-7 bound weakly to GST-AR-CTF and GST-EPR-CTF (Fig. 2C, panel 5). Lastly, we tested whether ZF5-8 is a critical region for interacting with AR-CTF or EPR-CTF. PLZF/ΔZnF5-8 bound none of the GST-CTFs (Fig. 2C, panel 6). These data suggest that the ZnF5-8 region is critical for the interactions between PLZF and the CTFs.

### Weak Binding Affinity of HB-EGF-CTF and PLZF was Important for the Nuclear Export of PLZF

**Figure 3:**
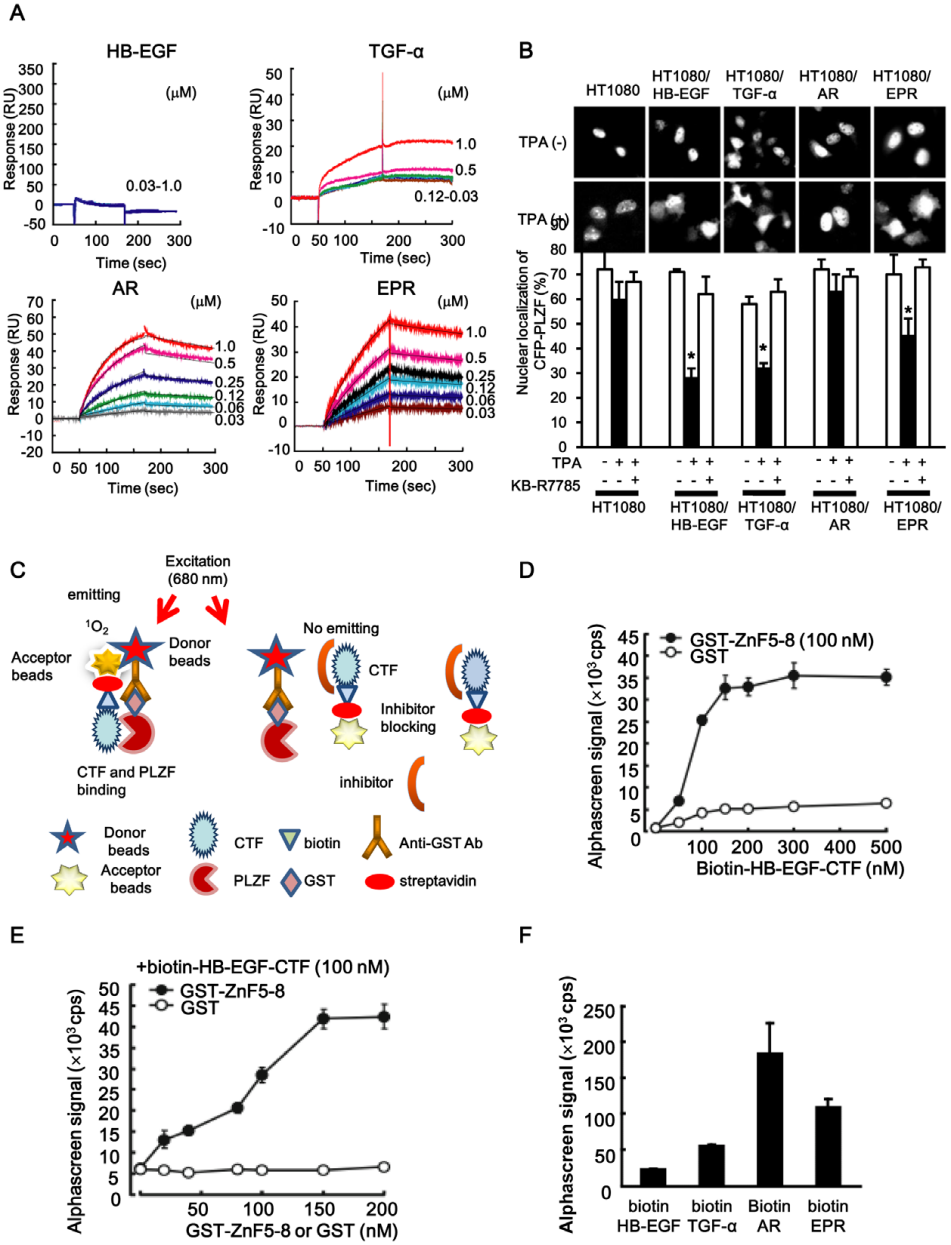
The interaction of EGFR-ligand-CTFs and PLZF, and high-throughput screening for inhibitors blocking the interaction. (A) Direct interaction between EGFR-ligand-CTF and PLZF (GST-tagged-ZnF5-8) with the SPR system. The recombinant biotin-EGFR- ligand-CTFs were individually immobilized, by SA sensor chips. GST-Zn5-8 with concentrations ranging from 0.03 to 1.0 µM was then injected with running buffer at 25°C and a flow rate of 30 µL/min for 2 min. (B) The expression vector encoding CFP-PLZF was co-transfected into wild-type HT1080 cells and HT1080 cells stably expressing either proHB-EGF, proTGF-α, proAR or proEPR. The cells were cultured for 24 h and then pretreated in serum-free medium with KB-R7785. Cells were then treated in the serum-free conditioned medium with TPA, and the subcellular localization of the CFP fusion protein was observed. To determine the percentage of cells (mean±SD) demonstrating nuclear localization of CFP-PLZF, cells were counted in at least two transfections, and at least 200 cells expressing CFP-PLZF were examined in each experiment. *P<0.05 for the stimulus effect during TPA treatment vs. no treatment, and **P<0.05 for the inhibitory effect during KB-R7785 treatment vs. TPA treatment. (C) A schematic of the high-throughput Alphascreen system. Upon excitation at 680 nm, ambient oxygen is converted to singlet oxygen (^1^O_2_) by a photosensitizer present in the donor beads. If the acceptor beads are in close proximity (<200 nm), ^1^O_2_ transfers its energy to thioxene derivatives present in the acceptor beads leading to emission of light at 520–620 nm. One complex consists of streptavidin-coated donor beads and biotinylated EGFR ligands-CTF. Another consists of anti-GST antibody-conjugated acceptor beads and GST-ZF5-8. If the association between EGFR ligand-CTF and ZnF5-8 occurs, the beads are close enough to allow detection of a signal. Any inhibitors of this interaction would increase the distance between the beads, and the signal would be lost. (D) Alphascreen signals of GST or GST-Zn5-8 incubated with various concentrations of biotin-HB-EGF-CTF. (E) Alphascreen signals of biotin-HB-EGF-CTF incubated with various concentrations of GST or GST-Zn5-8. (E) The binding abilities of HB-EGF, TGF-α, amphiregulin (AR), and epiregulin (EPR), to PLZF with Alphascreen system.

To further clarify whether the four CTFs directly interact with PLZF, we analyzed the binding between the CTFs and recombinant GST-tagged ZnF5-8 (GST-ZnF5-8) using a SPR system. GST-ZnF5-8 bound with immobilized biotin-AR-CTF and biotin–EPR-CTF (Fig. 3A) at a concentration of 0.03–1.0 µM. The *K*_D_ for the interaction between GST-ZnF5-8 and biotin-AR-CTF was 76.5 nM, and for GST-ZnF5-8 and biotin-EPR-CTF was 146 nM (Table 1). In contrast, the *K*_D_ value for the interaction between GST-ZnF5-8 and biotin-HB-EGF-CTF was not calculated due to their rapid dissociation. The binding signal of GST-ZnF5-8 to immobilized biotin-TGF-α-CTF was observed, but it was lower than that of biotin-AR-CTF or biotin-EPR-CTF. Thus, the dissociation constant could not be calculated because of the absence of dose-dependent binding of GST-ZF5-8 at a concentration of ≤0.5 µM and interference with the free SH residues of the seven cysteines on TGF-α-CTF (Fig. 3A).

**Table 1:** The direct interaction between Amphiregulin(AR), Epiregulin(EPR)-CTF and PLZF using surface plasmon resonance(SPR) spectroscopy.

	ka(M^−1^s^−1^)	kd(s^−1^)	KD(M)
AR-CTF	1.87×10^4^	1.44×10^−3^	7.65×10^−8^
EPR-CTF	5.61×10^3^	8.06×10^−4^	1.46×10^−7^

A direct interaction between EGFR-ligand-CTF and PLZF (GST-tagged-ZnF5-8) with the SPR system. Rate constants (ka) and dissociation constants (kd) for GST-ZnF5-8 binding to biotin-AR-CTF or biotin-EPR-CTF are performed. KD = kd/ka.

Next, to clarify nuclear export of PLZF triggered by the TPA-inducible shedding of EGFR ligands, the subcellular localization of the PLZF protein was examined during the intracellular translocation of the four CTFs. The expression vector encoding CFP-PLZF was transfected into HT1080 cells and the stably transfected HT1080 cells expressing either proHB-EGF, proTGF-α, proAR or proEPR. It is known that wild-type HT1080 cells constitutively express less HB-EGF and thus, the nuclear translocation of HB-EGF-CTF is not observed [14]. CFP-PLZF was predominantly localized in the nucleus of HT1080 cells and in the four transfectants. TPA treatment for 1 h induced a distribution of CFP-PLZF throughout the entire cytoplasm of HT1080/HB-EGF, HT1080/TGF-α, and HT1080/EPR cells. However, TPA did not alter the subcellular localization of CFP-PLZF in HT1080 and HT1080/AR cells. The ratios of CFP-PLZF localizing in the nucleus after TPA-inducible shedding were significantly low in HT1080/HB-EGF, HT1080/TGF-α and HT1080/EPR cells (Fig. 3B). Pre-treatment with 10 µM of KB-R7785, a metalloproteinase inhibitor, effectively abrogated the frequency of CFP-PLZF nuclear export after TPA stimulation in HT1080/HB-EGF, HT1080/TGF-α and HT1080/EPR cells (Fig. 3B). Intriguingly, the frequency of the nuclear export of PLZF is inversely correlated with its affinity. These results indicate that the affinity of CTF-PLZF interaction affects intracellular localization and function of PLZF.

### High-throughput Screening Assay System for the Inhibitors that Block the Interaction between the ZnF5-8 of PLZF and AR- or HB-EGF-CTF Narrowed 12 Candidates and Four ARBs

A high-throughput screening assay was developed on an Alphascreen® to screen for inhibitor that block the interaction between ZnF5-8 and CTFs. The Alphascreen assay design relies on the fact that a signal can be detected only when streptavidin-coated donor and anti-GST antibody-conjugated acceptor beads are closed within a distance of 200 nm. The beads are brought into close proximity for this reaction via specific interactions of ZnF5-8 and CTFs complexes coupled to them (Fig. 3C). Increasing concentrations of biotin-HB-EGF-CTF dose-dependently increased the Alphascreen signal, when incubated with GST-ZnF5-8 (Fig. 3D). Increasing concentrations of GST-ZnF5-8 also dose-dependently increased the signal when incubated with biotin-HB-EGF-CTF (Fig. 3E). The Alphascreen signals for the interaction between biotin-AR-CTF, biotin-EPR-CTF, biotin-TGF-α-CTF, and biotin-HB-EGF-CTF with GST-ZnF5-8 were comparable to the binding affinities determined with SPR system (Fig. 3F).

**Figure 4:**
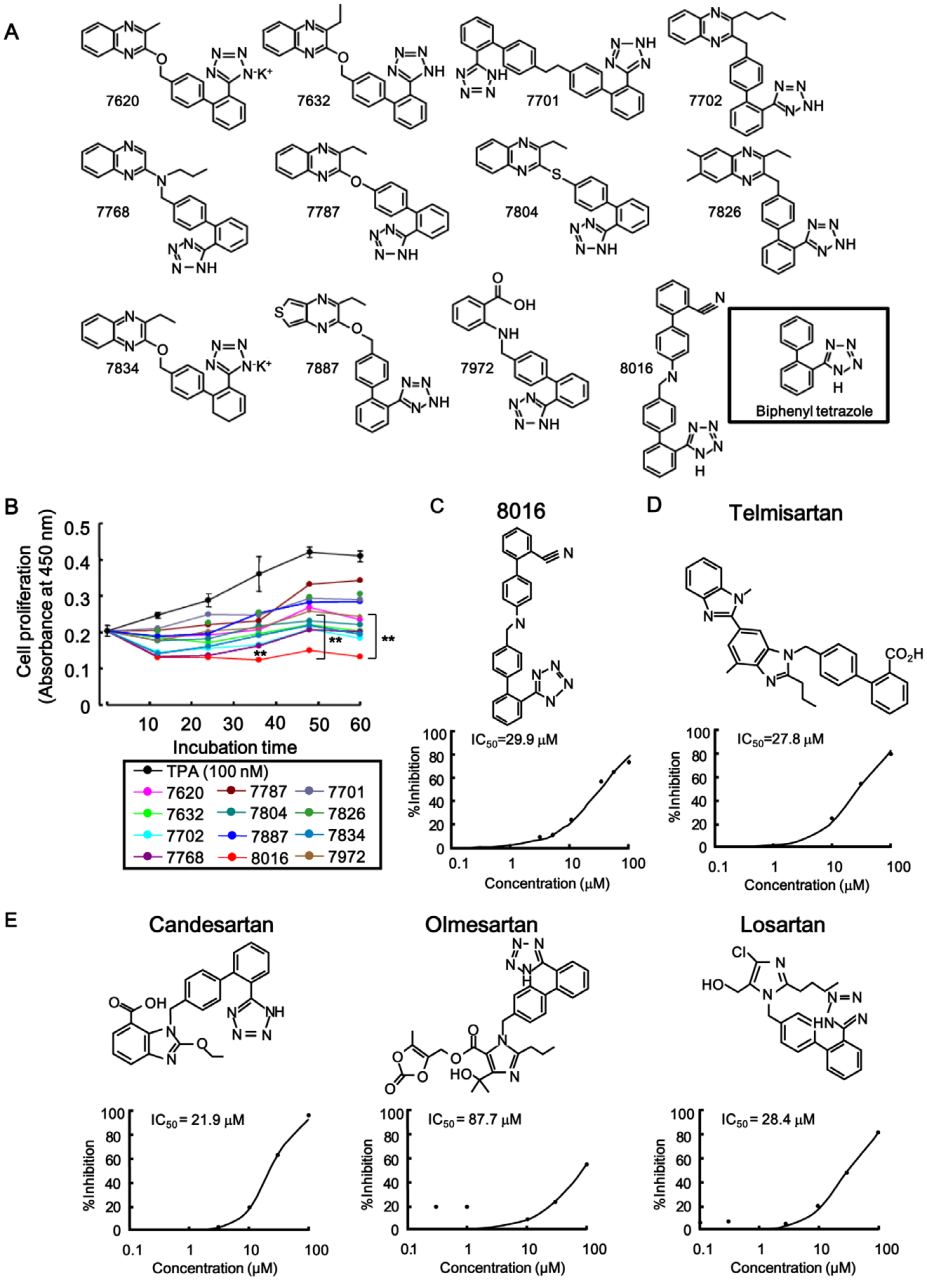
The potent candidates as inhibitors and their inhibitory effects on cell proliferation. (A) Twelve candidate compound’s structural formula based on efficacy for blocking binding of HB-EGF-CTF or AR-CTF to Zn5-8 of PLZF with Alphascreen system. (B) Inhibitory effects of twelve candidate compounds on TPA-induced cell proliferation in keratinocytes with a cell proliferation assay. 2×10^4^ cells were treated in the conditioned media with or without the twelve candidate compounds during TPA stimulation, and absorbance at 450 nm was determined every 12 h till 60 h. **P<0.05 for the inhibitory effects during 12 candidates treatment vs. TPA treatment. (C, D) The inhibitory effects of the candidates, specifically compound no.8016 and telmisartan, on the binding of AR-CTF to PLZF. Plots with the percent inhibition against various concentrations of compound no.8016 and telmisartan presented with the IC_50_ values observed by Alphascreen system. (E) Inhibitory effects of three ARB candidates on the binding of AR-CTF to PLZF. Plots with the percent inhibition against various concentrations of each inhibitor and inhibition presented with IC_50_ values.

An analysis with biotin-AR-CTF and GST-Zn5-8 of PLZF could clearly indicate inhibitory effects of obtained candidates on interaction of biotin-AR-CTF with GST-Zn5-8 of PLZF because Alphascreen signal of biotin-AR-CTF was higher than biotin-HB-EGF-CTF. Based on their efficacy of blocking the binding of AR-CTF as well as HB-EGF-CTF and to Zn5-8 of PLZF, twelve candidates, including no.8016, 7701 and 7804 were obtained by screening 9000 chemical compounds (Fig. 4A). The % inhibition of 10 µM of no. 8016, 7701 and 7804 on the interaction between biotin-AR-CTF and GST-Zn5-8 of PLZF were 30.4, 60.5 and 49.9, respectively. The inhibitory effect of compound no.8016 on the interaction was not the highest (Table 2). However, No.8016 inhibited cell proliferation strongest among twelve candidates in cell proliferation assay (Fig. 4B). Based on the results of cell proliferation, we selected no. 8016 as a candidate. The half maximal inhibitory concentration (IC_50_) obtained with compound no. 8016 was 29.9 µM (Fig. 4C).

**Table 2:** High-throughput screening (Alphascreen^®^) system for inhibitors that block the interaction between the PLZF and HB-EGF- or AR-CTF.

	Biotin-HB-EGF-CTF		Biotin-AR-CTF	
	100 µM	10 µM	100 µM	10 µM
	%Inhibition		%Inhibition	
No.7620	82.1	22.1	76.3	25.6
No.7632	81.7	12.6	74.9	40.5
No.7701	89.6	41.6	71.5	60.5
No.7702	86.7	34.4	77.9	46.3
No.7768	73	24.1	53.2	32.6
No.7787	82.1	10	82.2	14.9
No.7804	85.7	19.5	79.8	49.9
No.7826	82.3	14.5	82.6	30.7
No.7834	90.7	21.5	77.1	43.6
No.7887	93.9	28.4	90.9	39.5
No.7972	67.5	9.9	70.1	34.2
No.8016	71.4	33.1	75	30.4
Telmisartan	*	*	66.9	48.5
Camdesartan	78.1	12.6	84.1	23.2
Olmesartan	*	*	51.8	20.5
Losartan	*	*	68.4	12.9

Compounds: 9,000 → 12, Z’-factor: 0.73±0.091, S/B: 20.6,

* not calculated.

Twelve candidate and four angiotensin II type 1 receptor blocker (ARB) based on efficacy for blocking binding of HB-EGF-CTF or AR-CTF to Zn5-8 of PLZF with Alphascreen system. Z’-factor showed to be 0.73 (0.5<Z’<1.0), suggesting the reliable data.

We then focused on the specific structural formula of the biphenyl tetrazole in the candidate compound, and further screened chemical compounds, including ARBs (i.e. telmisartan, candesartan, olmesartan, and losartan). Based on the efficacy of blocking the binding of AR-CTF to PLZF, the IC_50_ of telmisartan, candesartan, olmesartan, and losartan were 27.8, 21.9, 87.7 and 28.4 µM, respectively, and their chemical structural formula are shown (Fig. 4D and E). These findings suggest that telmisartan and candesartan are satisfactory candidates in blocking both HB-EGF-CTF and AR-CTF from interacting with PLZF.

### No.8016 of Twelve Candidate Compounds and Telmisartan Inhibited Cell Proliferation

**Figure 5:**
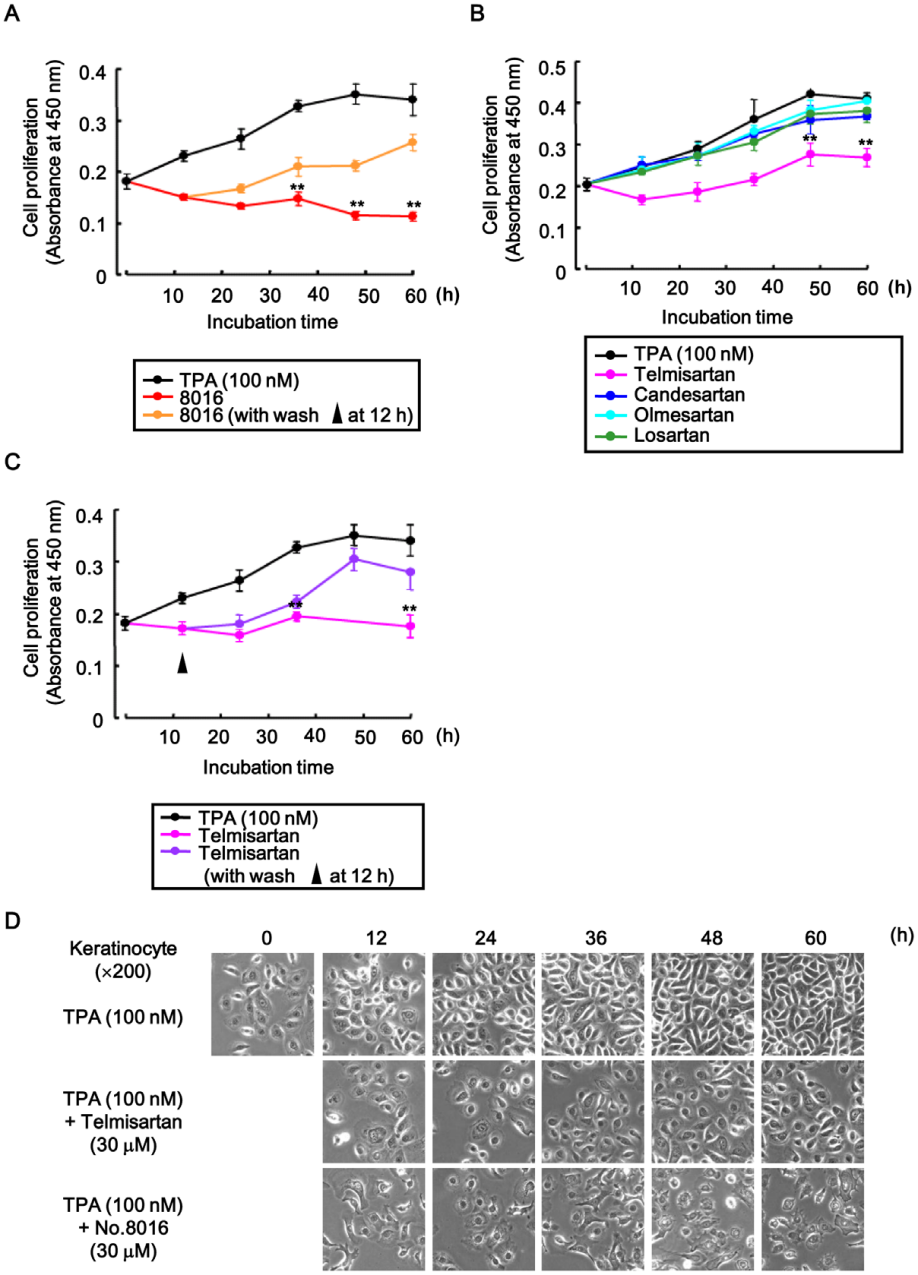
Inhibitory effects of no.8016 and four ARBs on cell proliferation. (A-D) Inhibitory effects of compound no.8016 (A) and four ARBs (B) on TPA-induced cell proliferation in keratinocytes with a cell proliferation assay. 2×10^4^ cells were treated in conditioned media with or without the no.8016 or four ARBs during TPA stimulation, and absorbance at 450 nm was determined every 12 h till 60 h. (A, C) Inhibition of cell proliferation with no.8016 and telmisartan was verified following a wash at 12 h. (D) The cells were also observed with microscopy. **P<0.05 for the inhibitory effects during 12 candidates and four ARBs treatment vs. TPA treatment.

Keratinocyte cells have a lot of endogenous HB-EGF precursors, and they are helpful to analyze cell proliferation through HB-EGF-CTF signaling. We used the HT1080 cell lines when inhibitors were screened [17]. To examine whether the candidates compounds that blocked the interaction between the CTFs and PLZF also inhibited cell proliferation, we tested the inhibitory effects of these twelve candidates and four angiotensin II type 1 receptor (AT1R) blockers (ARBs) on TPA-induced cell proliferation of keratinocyte with a cell proliferation assay. Cells were treated in conditioned media with or without 30 µM of the twelve candidate compounds and 30 µM of ARBs for up to 60 h in the presence of TPA, and the absorbance was measured every 12 h. Compound no.8016 and telmisartan were found to inhibit cell proliferation (Fig. 4B, 5B and D). Furthermore, absorbance values gradually recovered those of TPA-induced levels (i.e. without compound no.8016 and telmisartan), when cells were washed 12 h after incubation with compound no.8016 and telmisartan (Fig. 5A and C).

### TPA-induced Cell Proliferation was Regulated through Dual Signaling Pathways

**Figure 6:**
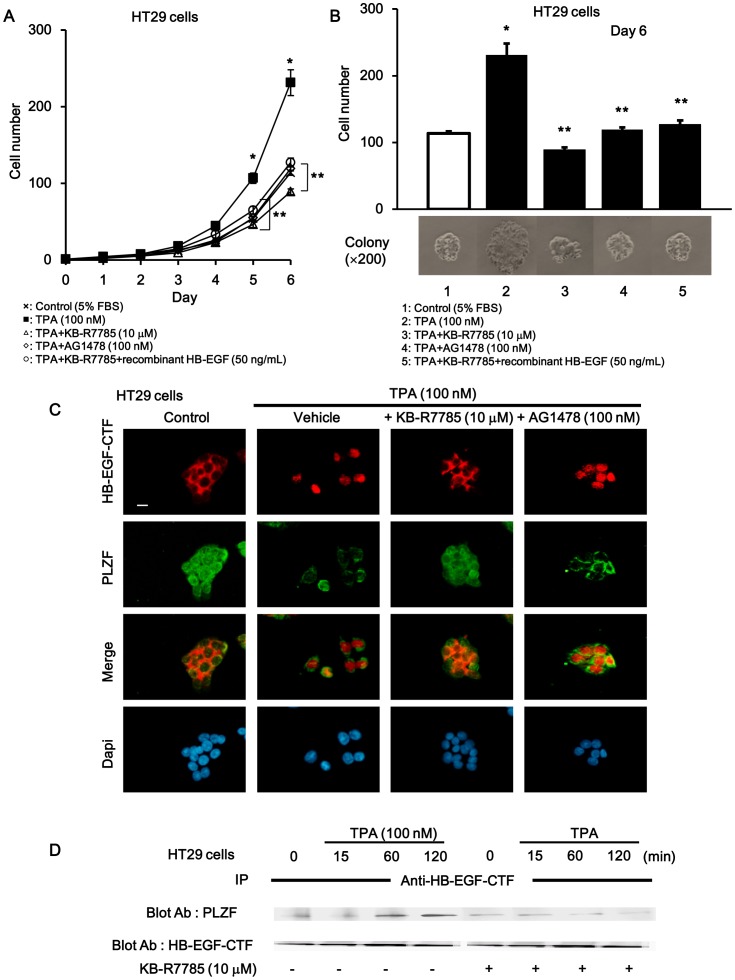
Cell proliferation through EGFR pathway and nuclear translocation of HB-EGF-CTF during TPA stimulation. TPA-induced cell proliferation through EGFR and nuclear translocation of HB-EGF-CTF signaling. (A) Growth curve assay. HT29 cell numbers which were counted daily, 24 h (i.e.day1) after cells were seeded in three dependent colonies that were cultured in conditioned media. The values are means of three independent experiments. (B) Cell numbers of colonies cultured in 5% FBS conditioned media with or without TPA, KB-R7785, AG1478, and recombinant HB-EGF on day6. The cells were also observed with microscopy (×200) *P <0.05 for the stimulus effect, and **P<0.05 for the inhibitory effect. (C) Effects of KB-R7785 and AG1478 on TPA-induced nuclear translocation of HB-EGF-CTF and nuclear export of PLZF. Cells were treated with TPA following preincubation with or without KB-R7785 and AG1478. Immunofluorescent staining with anti-HB-EGF-CTF antibodies (red signals), anti-PLZF antibodies (green) and DAPI (blue), which stains for nuclei was performed. Images were obtained on a fluorescence microscope (×200). The white bar indicated 10 µm. (D) Effects of KB-R7785 on the association between HB-EGF-CTF and PLZF after TPA stimulation. Cells were treated with TPA at various times following preincubation with or without KB-R7785. Blotted samples were probed with antibodies against PLZF after immunoprecipitation with anti-HB-EGF-CTF antibody (*upper panel*). The total amount of HB-EGF-CTF in the immunoprecipitates was determined by reprobing the same blot with an anti-HB-EGF antibody (*lower panel*).

We next verified that EGFR phosphorylation and nuclear translocation of HB-EGF-CTF signaling promoted cell proliferation. TPA is one of the activators of protein kinase C (PKC) δ and induces the ectodomain cleavage of proHB-EGF via ADAM [10]. When HT29 cells were continuously cultured in the presence or absence of TPA with or without KB-R7785, EGFR tyrosine kinase inhibitor AG1478, and recombinant HB-EGF (Fig. 6A), there was a 2-fold increase in cell counts with TPA compared with control (Fig. 6B, lane 2). To investigate whether ADAM mediated the TPA-promoted cell proliferation, we tested the effects of KB-R7785 on TPA-induced cell proliferation. KB-R7785 was used to block the shedding of the EGFR ligands and subsequent nuclear translocation of EGFR ligand-CTF. The TPA-induced increase in cell counts was completely blocked by KB-R7785, where the cells count was similar to the control levels seen on day 6 (Fig. 6B, lane 3). To investigate whether EGFR activation mediated the TPA-induced cell proliferation, we tested the effects of AG1478 on TPA-induced cell proliferation. The increase in cell counts induced by TPA was completely blocked by AG1478 to control levels obtained on day 6 (Fig. 6B, lane 4). To investigate whether nuclear translocation of HB-EGF-CTF mediates TPA-induced cell proliferation, we also tested the effects of recombinant HB-EGF on TPA-induced cell proliferation blocked by KB-R7785. KB-R7785 blocked TPA-induced cell proliferation even with EGFR activation via recombinant HB-EGF (Fig. 6B, lane 5). EGFR activation with recombinant HB-EGF during inhibition of EGFR ligand shedding with KB-R7785 did not recover cell proliferation to the levels achieved with TPA-stimulation. These data suggests that nuclear translocation of HB-EGF-CTF is the predominant player involved in cell proliferation.

Then, to examine the effects of KB-R7785 and AG1478 on the TPA-induced nuclear translocation of HB-EGF-CTF, we investigated the localization of HB-EGF-CTF and PLZF during TPA stimulation with or without KB-R7785 or AG1478 in HT29 cells with immunofluorescent staining (Fig. 6C). In control (i.e. serum-free), HB-EGF-CTF was located in the cell membrane or cytoplasm, and PLZF was located in the nucleus. Nuclear translocation of HB-EGF-CTF and nuclear export of PLZF was evident during TPA stimulation. KB-R7785 blocked the TPA-induced nuclear translocation of HB-EGF-CTF and nuclear export of PLZF. However, AG1478 did not inhibit these TPA-dependent effects.

We also conformed whether TPA induced the association of HB-EGF-CTF to PLZF. After cultured cells were treated with TPA in the presence or absence of KB-R7785, the association between HB-EGF-CTF and PLZF was assessed by immunoprecipitation with the anti-HB-EGF-CTF antibody, followed by Western blotting with the anti-PLZF antibody. The association between HB-EGF-CTF and PLZF following TPA stimulation peaked at 120 min. KB-R7785 completely blocked this association (Fig. 6D).

### Telmisartan, but not Candesartan, Inhibited TPA-induced Colon Cancer Cell Proliferation

**Figure 7:**
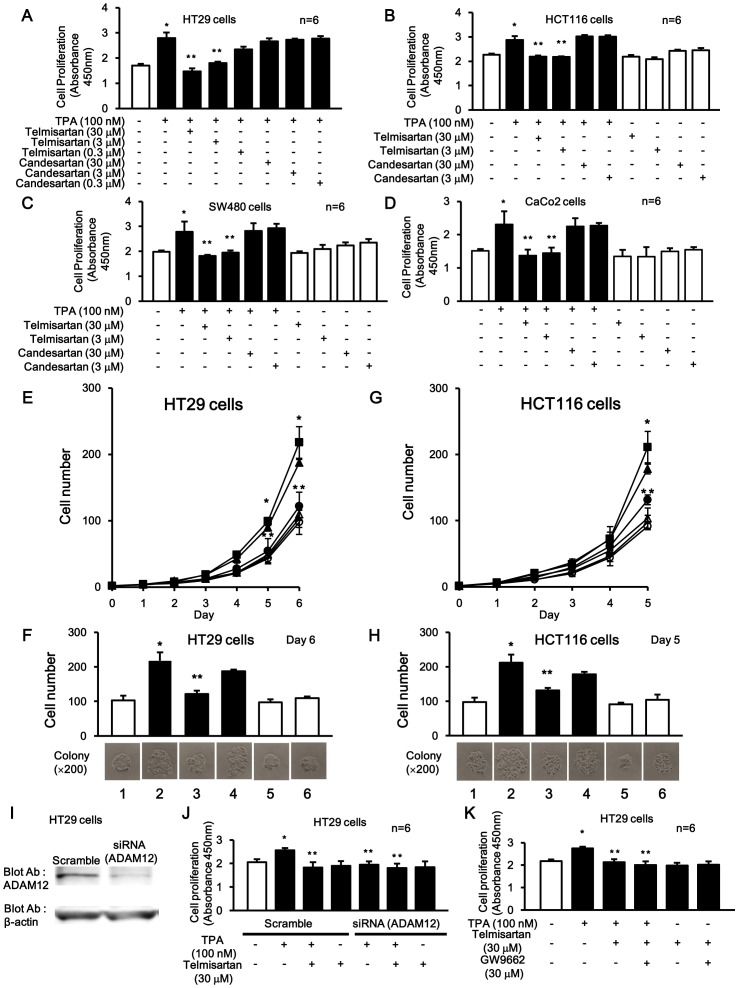
The inhibitory effects of telmisartan and candesartan on TPA-induced cell proliferation in colon cancer cells. (A-D) Inhibitory effects of telmisartan, but not candesartan, on TPA-induced cell proliferation in HT29 cells (A), HCT116 (B), SW480 (C), and CaCo2 (D) with CCK-8 kit assay. After 24 h of plating, 2×10^5^ cells were incubated for 72 h in conditioned media with or without telmisartan, candesartan and/or TPA. Each bar represents the means of six independent experiments. *P<0.05 for the stimulus effect, and **P<0.05 for the inhibitory effect. (E and G) In growth curve assay, HT29 and HCT116 cell numbers were counted daily in three dependent colonies cultured in conditioned media. The values are means of three independent experiments. ×; Control (5%FBS), ▪; TPA (100 nM), ○; telmisartan (30 µM), •; TPA+telmisartan (30 µM), △; candesartan (30 µM), ▴; TPA+candesartan (30 µM). (F and H) Cell numbers in colonies cultured in conditioned media with or without TPA, telmisartan and candesartan on day 5 or 6. *P<0.05 for the stimulus effect, and **P <0.05 for the inhibitory effect. Lane 1; Control (5% FBS white box), 2; TPA (100 nM), 3; TPA+telmisartan (30 µM), 4; TPA+candesartan (30 µM), 5; telmisartan (30 µM), 6; candesartan (30 µM). (I) Knockdown of ADAM12 with siRNA. Probing with an anti-ADAM12 antibody (upper panel) and anti- β-actin antibody (lower panel). (J) Inhibitory effects of telmisartan on TPA-induced cell proliferation in HT29 cells with CCK-8 kit assay following knockdown of ADAM12 with siRNA. *P<0.05 for the stimulus effect, and **P <0.05 for the inhibitory effect. (K) Inhibitory effects of telmisartan and GW9662 on TPA-induced cell proliferation in HT29 cells with CCK-8 kit assay. *P<0.05 for the stimulus effect, and **P<0.05 for the inhibitory effect.

Telmisartan inhibited TPA-induced cell proliferation in a dose-dependent manner, but not candesartan (Fig. 7A) in a CCK-8 cell proliferation assay on HT29 cells. And, inhibitory effects of 3 µM and 30 µM with telmisartan on this cell proliferation were also observed in other colon cancer cell lines (HCT116, SW480, and CaCo2) (Fig. 7B–D).

In addition, the inhibitory effects of 30 µM of telmisartan and candesartan on TPA-induced cell proliferation were assessed with a growth curve analysis and cell proliferation assay. HT29 (Fig. 7E and F) and HCT116 (Fig. 7G and H) cells were continuously cultured for up to 5 or 6 days in the presence or absence of TPA with or without telmisartan and candesartan. The cell counts were 2-fold greater with TPA stimulation compared to control (Fig. 7F and H, lane 2). These TPA-induced cell counts were inhibited by telmisartan (Fig. 7F and H, lane 3), but not candesartan (Fig. 7F and H, lane 4) to approximately control levels. However, in the absences of TPA stimulation, both telmisartan and candesartan (Fig. 7F and H, lane 5–6) did not further inhibit cell proliferation (i.e. below control levels).

It is known that telmisartan has an anti-cancer activity as a PPARγ agonist [20], [21]. ADAM12 reportedly mediated TPA-induced HB-EGF shedding and subsequently nuclear translocation of HB-EGF-CTF [14]. Next, to clearly investigate the PPARγ effect of telmisantan during elimination of TPA-induced cell proliferation signal by depleting ADAM12, we examined cell proliferation in the presence or absence of telmisartan during TPA stimulation with or without ADAM12 depletion (Fig. 7I and J). ADAM12 depletion inhibited TPA-induced cell proliferation. Moreover, an additively inhibitory effect of telmisartan was little observed. In addition, we examined whether the inhibitory effect of telmisartan on cell proliferation was cancelled by using GW9662. Telmisartan inhibited TPA-induced cell proliferation. Moreover, an additively inhibitory effect of telmisartan was little observed (Fig. 7K).

### Nuclear Translocation of HB-EGF-CTF and Nuclear Export of PLZF, and the Association with HB-EGF-CTF to PLZF, during TPA Stimulation were Blocked by Telmisartan, but not by Candesartan

**Figure 8:**
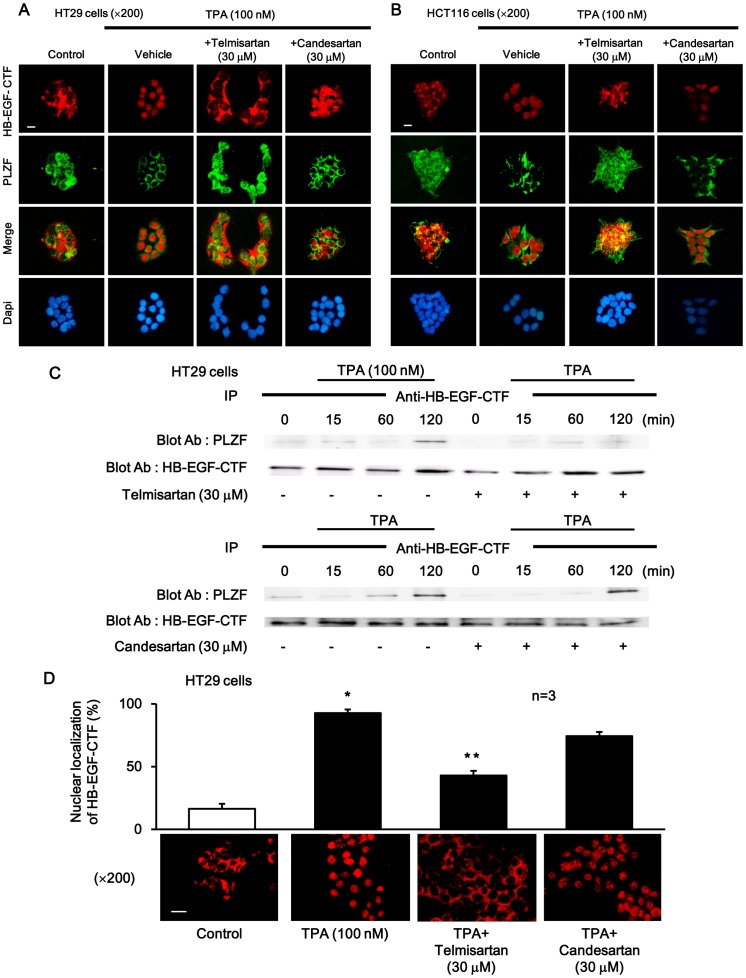
The effects of telmisartan or candesartan on localization and binding of HB-EGF-CTF and PLZF. (A and B) Immunofluorescent stainings with anti-HB-EGF-CTF antibodies (red), anti-PLZF antibodies (green), and DAPI (blue), in the presence or absence of TPA after pretreatment with telmisartan or candesartan. Images were obtained on a fluorescence microscope (×200). The white bar indicated 10 µm. (C) Effects of telmisartan or candesartan on the TPA-induced association between HB-EGF-CTF and PLZF. HT29 cells were preincubated with or without telmisartan or candesartan. The cells were then treated with TPA for 0, 30, 60 and 120 min. Blotted samples were probed with an anti-PLZF antibody after immunoprecipitation with the anti-HB-EGF-CTF antibody (upper panel). The total amount of HB-EGF-CTF in the immunoprecipitates was determined by reprobing the same blot with an anti-HB-EGF-CTF antibody (lower panel). (D)Three staining regions were randomly selected under a ×200 field and the total for three regions was calculated as the positive rate of cells displaying nuclear staining. The percentage of cells showing nuclear staining is shown for 100 nM of TPA, and 30 µM of telmisartan and candesartan. Images were obtained on a fluorescence microscope (×200). The white bar indicated 10 µm. *P<0.05 for the stimulus effect, and **P<0.05 for the inhibitory effect.

Immunofluorescent staining was performed in HT29 or HCT116 cells to determine the intracellular localization of HB-EGF-CTF and PLZF during TPA stimulation with or without telmisartan or candesartan. Cultured cells were treated with TPA after incubation with either telmisartan or candesartan for 1 h. HB-EGF-CTF was located in the cell membrane or cytoplasm, and PLZF in the nucleus, in non-stimulated control cells. Nuclear translocation of HB-EGF-CTF and nuclear export of PLZF were seen during TPA stimulation. Telmisartan, but not candesartan, inhibited the TPA-induced nuclear translocation of HB-EGF-CTF and nuclear export of PLZF (Fig. 8A and B).

We also confirmed whether TPA induced the association between HB-EGF-CTF and PLZF in HT29 cells. The association between both was assessed by immunoprecipitation with the anti-HB-EGF-CTF antibody, followed by Western blotting with the anti-PLZF antibody, in cultured cells treated with TPA in the presence or absence of telmisartan or candesartan. The association peaked at 120 min. Telmisartan, but not candesartan, significantly blocked this association (Fig. 8C).

The percentage of cells displaying nuclear staining for HB-EGF-CTF was significantly decreased when HT29 cells were incubated via TPA with pretreatment of 30 µM of telmisartan, compared to pretreatment of 30 µM of candesartan (Fig. 8D).

### Telmisartan AT1R-independently Inhibited Nuclear Translocation of HB-EGF-CTF and Cell Proliferation

**Figure 9:**
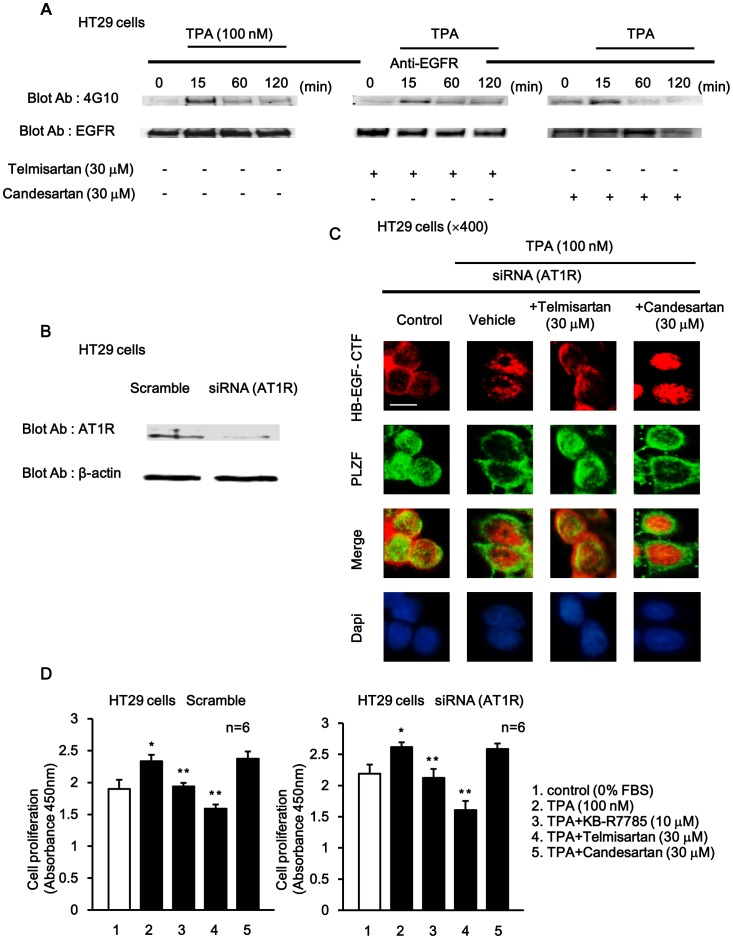
The effects of telmisartan and candesartan on HB-EGF-CTF nuclear translocation and cell proliferation during AT1R depletion. (A) Effects of telmisartan or candesartan on TPA-induced EGFR phosphorylation. Cells were preincubated with or without telmisartan or candesartan, and then treated with TPA for 0, 15, 60 and 120 min. Blotted samples were probed with an anti-phosphotyrosine antibody after immunoprecipitation with an anti-EGFR antibody (*upper panel*). The total amount of EGFR in the immunoprecipitates was determined by reprobing the same blot with an anti-EGFR antibody (*lower panel*). (B) Effects of telmisartan or candesartan on TPA-induced nuclear translocation of HB-EGF-CTF and nuclear export of PLZF following knockdown of AT1R with siRNA. Probing with an anti-AT1R antibody (*upper panel*) and anti-β-actin antibody *(lower panel*). (C) Cells were then treated with TPA following preincubation with or without telmisartan or candesartan. Immunofluorescent stainings with anti-HB-EGF-CTF antibodies (red), anti-PLZF antibodies (green) and DAPI (blue) were performed following knockdown of AT1R with siRNA. Images were obtained on a fluorescence microscope (×400). The white bar indicated 10 µm. (D)The inhibitory effects of telmisartan and candesartan on TPA-induced cell proliferation in HT29 cells with CCK-8 kit assay following knockdown of AT1R with siRNA. *P<0.05 for the stimulus effect, and **P<0.05 for the inhibitory effect.

To assess the effect of telmisartan and candesartan on EGFR phosphorylation, EGFR phosphorylation was assessed by immunoprecipitation using an anti-EGFR antibody followed by Western blotting with an anti-phosphotyrosine antibody. Cells incubated with or without telmisartan or candesartan were then treated with TPA at various times. EGFR phosphorylation following stimulation with TPA peaked within 15 min and then gradually decreased to basal levels within 120 min. EGFR phosphorylation was not inhibited by either telmisartan or candesartan (Fig. 9A).

Next, both telmisartan and candesartan are antagonists of the AT1R. And, to determine whether the TPA-induced nuclear translocation of HB-EGF-CTF and nuclear export of PLZF are independent of AT1R signaling, the intracellular localization of HB-EGF-CTF and PLZF was confirmed after knocking down the AT1R with short interfering RNA (siRNA). First, a complete knockdown of endogenous AT1R protein was achieved with siRNA (Fig. 9B). Then, HT29 cells were pre-treated with telmisartan or candesartan, stimulated with TPA, and immunofluorescent staining with anti-HB-EGF-CTF and anti-PLZF antibodies was then performed. In control cells, HB-EGF-CTF was found in the cell membrane or cytoplasm, and PLZF was done in the nucleus. Nuclear translocation of HB-EGF-CTF and nuclear export of PLZF were evident during TPA stimulation. Telmisartan, but not candesartan, inhibited TPA-induced nuclear translocation of HB-EGF-CTF and nuclear export of PLZF, even after treatment during AT1R depletion (Fig. 9C).

Finally, we tested the effects of KB-R7785, telmisartan, and candesartan on TPA-induced cell proliferation after AT1R depletion with siRNA in HT29 cells with the CCK-8 cell proliferation assays. The TPA-induced cell proliferation was inhibited by KB-R7785, and telmisartan, but not by candesartan in the presence and absence of AT1R depletion (Fig. 9D).

## Discussion

We have here shown that TPA promoted cell proliferation through nuclear translocation signaling of HB-EGF-CTF, and that telmisartan blocked the binding of HB-EGF-CTF to PLZF, which in turn, significantly inhibited cell proliferation.

First, we screened for an inhibitor that was capable of blocking the interaction between HB-EGF-CTF and PLZF. We previously showed that HB-EGF-CTF was associated with the transcriptional repressor, PLZF, and triggered its nuclear export [12]. A GST pull-down assay assessing the *in vitro* interaction between PLZF and four CTFs, namely TGF-α-CTF, AR-CTF, EPR-CTF, and HB-EGF-CTF, demonstrated that PLZF interacted with all four CTFs. However, the deletion mutant, PLZF/ΔZnF5-8, did not bind any of the CTFs. These data suggest that the ZnF5-8 region is critical for the interactions between PLZF and the CTFs. Moreover, SPR analysis revealed that the binding affinities of ZnF5-8 for AR-CTF and EPR-CTF were 76.5 nM and 146 nM, respectively, which were higher than those of either HB-EGF-CTF or TGF-α-CTF (Fig. 3A). Immunostaining of the TPA-trigged PLZF nuclear export demonstrated that PLZF was localized with in the cytoplasm of HT1080/HB-EGF, HT1080/TGF-α and HT1080/EPR cells, but not in the HT1080/AR cells (Fig. 3B). These suggested that AR bound PLZF more strongly than HB-EGF in the nucleus, but that AR did not feasibly release the binding in the cytoplasm than HB-EGF. Based on these observations, the inverse correlation between binding affinity and nuclear export were evident. Thus, the interaction between HB-EGF-CTF and PLZF in the nucleus followed by the rapid release of PLZF from HB-EGF-CTF in the cytoplasm, appears to regulate its downstream signaling, and was therefore characterized as a key event during cell proliferation. The SPR system uses a highly specialized optical technique to analyze biomolecular interactions and provides both qualitative and quantitative date. Additionally, in the present study, we established a very useful assay system to cyclopaedically quantify the interactions between EGFR ligand-CTFs and ZnF5-8 of PLZF using Alphascreen (Fig. 3C). Given that the estimations of the interaction between EGFR ligand-CTFs and ZnF5-8 with Alphascreen were comparable to those obtained with the SPR analysis, Alphascreen was a useful and powerful for the high-throughput screening of compounds, which inhibited these interactions with EGFR ligand-CTFs and its partners, but we need to prepare short peptides with the specified binding sites between the both and manipulate binding of those peptides to beads. Thus we ended up using the Alphascreen methodology, and focused on screening compounds containing the specific structural formula of biphenyl tetrazole. This led us to identifying telmisartan and candesartan as potential candidates.

Subsequently, we attempted to characterize the predominant signaling pathway involved in the TPA-induced cell proliferation, specifically EGFR signaling or nuclear translocation of HB-EGF-CTF in HT29 cells. KB-R7785 was used to block both intracellular signaling pathway involved in cell proliferation. The growth curve assay demonstrated that KB-R7785 and AG1478 completely inhibited TPA-induced cell proliferation. Furthermore, EGFR activation with recombinant HB-EGF during inhibition of EGFR ligand shedding with KB-R7785 did not recover cell proliferation to the levels achieved with TPA-stimulation. This finding suggests that nuclear translocation of HB-EGF-CTF is the predominant player involved in cell proliferation. Furthermore, immunofluorescent staining and immunoprecipitation with the anti-HB-EGF-CTF antibody, followed by Western blotting with the anti-PLZF antibody, demonstrated that KB-R7785 completely blocked the nuclear translocation of HB-EGF-CTF, nuclear export of PLZF and the binding of HB-EGF-CTF to PLZF, during TPA stimulation. Thus nuclear translocation of HB-EGF-CTF also plays a central role on TPA-induced cell proliferation. These observations are consistent with the previous finding that HB-EGF-CTF on the cell surface translocate to the inner nuclear membrane [13], full-length forms of HB-EGF did not translocate to the nucleus in the gut cells overexpressing unshed HB-EGF-CTF [22], and the suppression of nuclear translocation of HB-EGF-CTF abrogated cell proliferation in gastric cancer cells [23]. We then tested whether both telmisartan and candesartan inhibited cell proliferation, nuclear translocation of HB-EGF-CTF and binding of HB-EGF-CTF to PLZF. Telmisartan, but not candesartan, significantly inhibited cell proliferation, nuclear translocation of HB-EGF-CTF and binding of HB-EGF-CTF to PLZF during TPA stimulation in HT29 and HCT116 cells (Fig. 7E–H). The differences in the inhibitory effects of telmisartan and candesartan on the abovementioned cellular function can be explained by their lipid solubility. Telmisartan is more lipid soluble than candesartan [24], and thereby, telmisartan can easily pass through the cell membrane into the cytoplasm and block the interaction between HB-EGF-CTF and PLZF.

Angiotensin II, a GPCR agonist, also induces EGFR transactivation [14]. In the present study, the TPA-induced nuclear translocation of HB-EGF-CTF and nuclear export of PLZF were independent of AT1R signaling during a knockdown of the AT1R (Fig. 9C). Further, TPA-induced cell proliferation was also independent of AT1R signaling (Fig. 9D). Based on these findings, telmisartan inhibited cell proliferation during TPA stimulation by specifically blocking nuclear translocation of HB-EGF-CTF AT1R-independently and nuclear transport of PLZF in colon cancer cells. In addition, there is no evidence of AT1R agonists that specifically block nuclear translocation of HB-EGF-CTF and binding of that to PLZF. We here discovered a new property of telmisartan.

A previous report demonstrated that telmisartan inhibited cell proliferation through EGFR followed by extracellular-regulated kinase (ERK) in uterine leiomyoma cells [25]. However, upstream molecular mechanism of EGFR signaling was unclear. In the present study, we could elucidate the inhibitory mechanism of telmisartan. In addition, telmisartan is a partial PPARγ agonist to activate PPARγ as the nuclear transcription factor [20]. PPARγ agonists induce apoptosis particularly in colon cancer cells and have inhibitory effects on cell proliferation [26]. Cell proliferation assays in the presence or absence of telmisartan during TPA stimulation with ADAM12 depletion and GW9662 showed that an additively inhibitory effect of telmisartan was little observed. It has also been demonstrated that PPARγ is a nuclear receptor of prostaglandins and leukotrienes as well, and it down-regulate cell proliferation in colon cancer cells [26]. These results and fact suggested that PPARγ signaling might not be associated with ADAM-HB-EGF-CTF signaling, and that inhibitory effect of telmisartan as PPARγ agonist on cell proliferation was weak during TPA stimulation.

A recent meta-analysis on the incidence of cancers during treatment with ARBs in15 large clinical trials found that there was no significant increase in lung, prostate or breast cancer risk and the overall cancer mortality, in patients receiving ARBs compared to controls [27]. In addition, telmisartan plus cetuximab inhibited cell proliferation in a dose-dependent manner, and better than telmisartan alone, as determined with cell proliferation assay (unpublished data). Thus, based on these findings, telmisartan plus combination chemotherapy with anti-EGFR therapy appears to be a better treatment strategy in patients with colorectal cancer, compared to combination chemotherapy with anti-EGFR therapy alone.

In conclusion, inhibition of HB-EGF-CTF nuclear translocation is important for blocking cell proliferation in colon cancer cell. telmisartan can inhibit cell proliferation by inhibiting the nuclear translocation of HB-EGF-CTF. Thus, telmisartan and its derivatives may be potential treatment strategies for the prevention of cell proliferation and colon cancer development.
